# Intermittent fasting attenuates lipopolysaccharide-induced neuroinflammation and memory impairment

**DOI:** 10.1186/1742-2094-11-85

**Published:** 2014-05-06

**Authors:** Andrea R Vasconcelos, Lidia M Yshii, Tania A Viel, Hudson S Buck, Mark P Mattson, Cristoforo Scavone, Elisa M Kawamoto

**Affiliations:** 1 Department of Pharmacology, Institute of Biomedical Science, University of São Paulo, São Paulo 05508-900, Brazil; 2 School of Arts, Sciences and Humanities, University of São Paulo, São Paulo 03828-080, Brazil; 3 Department of Physiological Sciences, Santa Casa de São Paulo Medical School, São Paulo 01221-020, Brazil; 4 Laboratory of Neurosciences, National Institute on Aging Intramural Research Program, Baltimore, MD 21224, USA

**Keywords:** Intermittent fasting, Inflammation, TLR4, Memory

## Abstract

**Background:**

Systemic bacterial infections often result in enduring cognitive impairment and are a risk factor for dementia. There are currently no effective treatments for infection-induced cognitive impairment. Previous studies have shown that intermittent fasting (IF) can increase the resistance of neurons to injury and disease by stimulating adaptive cellular stress responses. However, the impact of IF on the cognitive sequelae of systemic and brain inflammation is unknown.

**Methods:**

Rats on IF for 30 days received 1 mg/kg of lipopolysaccharide (LPS) or saline intravenously. Half of the rats were subjected to behavioral tests and the other half were euthanized two hours after LPS administration and the hippocampus was dissected and frozen for analyses.

**Results:**

Here, we report that IF ameliorates cognitive deficits in a rat model of sepsis by a mechanism involving NF-κB activation, suppression of the expression of pro-inflammatory cytokines, and enhancement of neurotrophic support. Treatment of rats with LPS resulted in deficits in cognitive performance in the Barnes maze and inhibitory avoidance tests, without changing locomotor activity, that were ameliorated in rats that had been maintained on the IF diet. IF also resulted in reduced levels of mRNAs encoding the LPS receptor TLR4 and inducible nitric oxide synthase (iNOS) in the hippocampus. Moreover, IF prevented LPS-induced elevation of IL-1α, IL-1β and TNF-α levels, and prevented the LPS-induced reduction of BDNF levels in the hippocampus. IF also significantly attenuated LPS-induced elevations of serum IL-1β, IFN-γ, RANTES, TNF-α and IL-6 levels.

**Conclusions:**

Taken together, our results suggest that IF induces adaptive responses in the brain and periphery that can suppress inflammation and preserve cognitive function in an animal model of systemic bacterial infection.

## Introduction

Systemic inflammation/sepsis is a risk factor for cognitive impairment and dementia [1]. The elderly are vulnerable to the adverse effects of infections on cognitive function, and the aging process itself is associated with increased neuroinflammatory processes involving microglial activation and production of pro-inflammatory cytokines [2,3]. Inflammation also occurs in association with the pathological changes in the brains of patients with Alzheimer’s disease (AD) and ischemic stroke [4]. However, clinical trials of anti-inflammatory therapies for AD, including NSAIDs, TNF inhibitors and intravenous immunoglobulin [5,6], have not been encouraging [7-10].

NF-κB, the activity of which is attributed to the Rel/NF-κB family proteins forming homo- and heterodimers through the combination of the subunits p65 (or RELA), p50, p52, cREL and RELB, can be activated by lipopolysaccharide (LPS), cytokines such as TNF-α and IL-1β, and reactive oxygen species [11,12]. NF-κB, which is constitutively expressed in the cytoplasm, is inhibited by a family of molecules termed inhibitor κB (IκBs). IκB binds NF-κB and masks its nuclear localization signal, thus retaining it in the cytoplasm [13]. Inducers of NF-κB act by intracellular signaling pathways that activate the IκB kinases (IKKs), which phosphorylate two specific N-terminal serines of IκBα, resulting in IκBα polyubiquitination and degradation in the 26S protease [14]. When IκB is degraded, NF-κB migrates to the nucleus, modulating the transcription of several genes associated with neurodegenerative or neuroprotective actions [15,16].

Bacterial infections activate innate immune signaling pathways involving toll-like receptor 4 (TLR4) and the transcription factor NF-κB in microglia and macrophages, which induces the expression of pro-inflammatory cytokines and the production of nitric oxide [17]. These pathways play critical roles in the killing and degradation of the bacteria by immune cells, but can also adversely affect neurons. Studies of TLR4-deficient mice suggest that TLR4 signaling has a negative impact on hippocampus-dependent cognitive function [18]. Data further suggest that TLR4 activation contributes to the degeneration of neurons in experimental models of AD [19,20] and stroke [21]. Lipopolysaccharide (LPS) is a major bacterial TLR4 ligand that activates the innate immune response to infection, and administration of LPS can cause cognitive impairment in animal models by mechanisms involving expression of pro-inflammatory cytokines and inhibition of neurotrophic factor production [22-26]. It is known that pro-inflammatory mediators disrupt hippocampal neuronal functions, including long-term potentiation and working memory consolidation [27,28]. Cytokines such as TNF-α, IL-1β and IL-6 are involved in hippocampal long-term potentiation and dendritic branching, which are processes involved in memory formation and maintenance [29]. In addition, it has already been shown that systemic administration of TNF-α reduces cell proliferation in the hippocampus, whereas no effect is observed with single doses of IL-1β and IL-6 [30].

IF can improve cognitive function in mouse models relevant to AD [31,32] and in old rats [33]. Also, IF reduces brain damage, increases levels of neurotrophic factors, and reduces markers of inflammation in a mouse model of focal ischemic stroke [34]. Here, we report that IF ameliorates cognitive deficits in a rat model of systemic bacterial infection, by a mechanism involving suppression of neuroinflammation and maintenance of neurotrophic support. Our findings suggest a potential for application of IF for the prevention and treatment of the cognitive deficits resulting from systemic inflammation.

## Material and methods

### Animals

**Figure 1:**
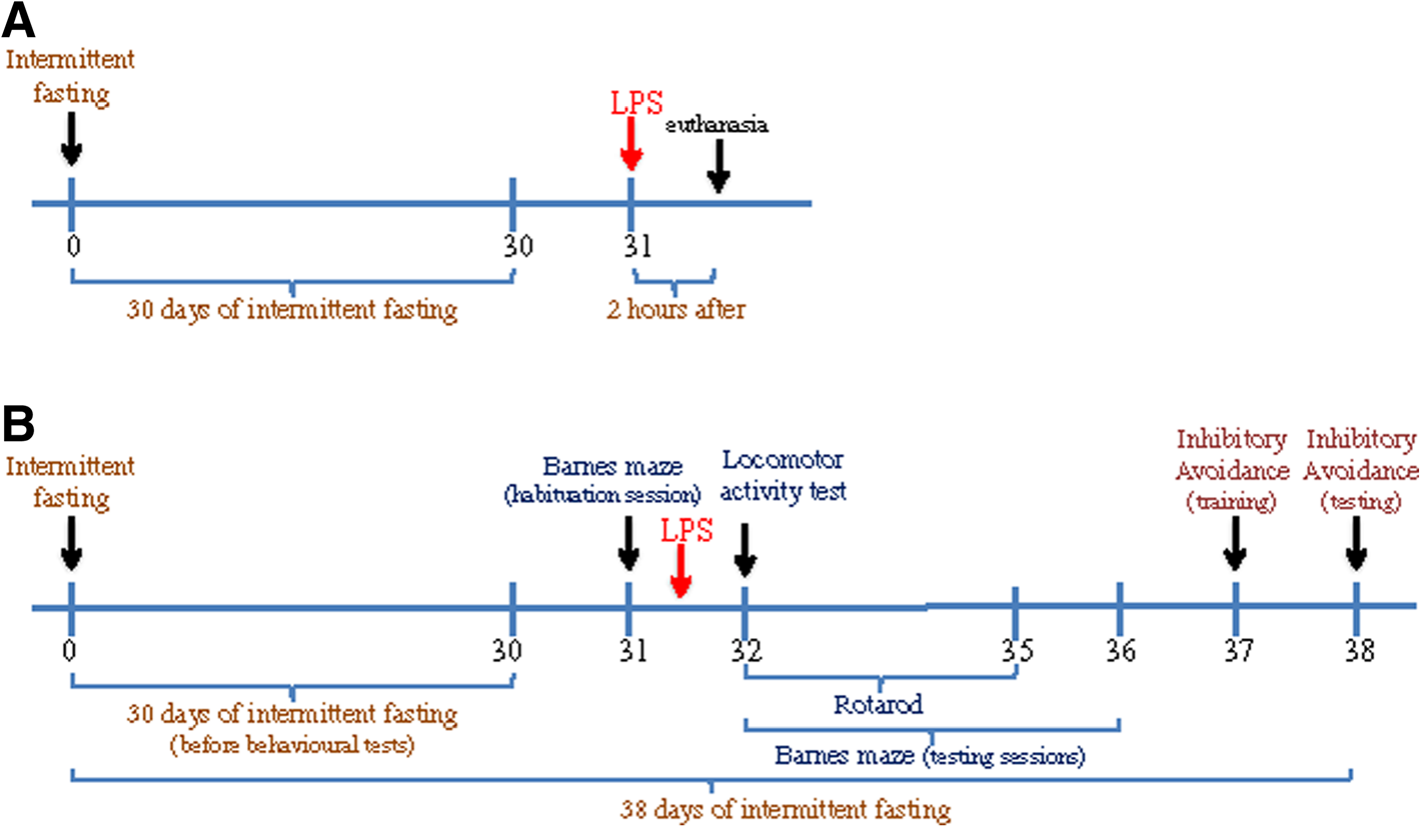
**Design of experiments to determine the effects of intermittent fasting (IF) on cognitive function and hippocampal neuroinflammation. (A)** For the biochemical experiments, rats were subjected to IF or *ad libitum* Control diets for 30 days. To determine whether IF modulated the inflammatory response to a subsequent challenge, rats were injected intravenously with either 1 mg/kg lipopolysaccharide (LPS) or saline on day 31, and 2 hours later brain tissue and serum samples were collected for subsequent analysis. **(B)** For the behavioral experiments, rats were subjected to the habituation session of the Barnes maze and were immediately injected intravenously with either 1 mg/kg LPS or saline on day 31. Barnes maze testing sessions and inhibitory avoidance tests were performed on days 32 to 36 and 37 to 38, respectively. Locomotor activity test was performed 24 hours after LPS administration and rotarod tests were performed on days 32 to 35.

Adult 12-week-old male Wistar rats were kept under a 12-hour light/12-hour dark cycle and allowed free access to water. All treatments were administered between 9:00 and 10:00 am. Rats were randomly assigned to a normal feeding group and an alternate-day fasting diet (IF). Rats on the IF diet were deprived of food for 24 hours every other day for 30 days. On the 31st day, after being given *ad libitum* access to food for 24 hours to avoid the effects of acute fasting, each animal received 1 mg/kg of LPS (O111:B4) (Sigma-Aldrich, St Louis, MO, USA) or saline intravenously [35]. Thus, four groups were used: saline (Control), LPS treatment (LPS), IF with saline (IF) and IF with LPS treatment (IF + LPS). The LPS challenge was performed approximately three hours after lights on (ranging from 09:00 am to 10:00 am). Half of the rats were subjected to behavioral tests, and the other half were euthanized two hours after LPS administration [35] and the hippocampus was dissected and frozen for analyses, according to the timelines shown in Figure 1. This research was approved by the Biomedical College of Animal Experimentation (COBEA). All procedures were also approved by the Ethical Committee for Animal Research (CEEA) of the Biomedical Sciences Institute of the University of São Paulo.

### Locomotor activity test

Spontaneous locomotor activity was measured using an electronic animal activity meter (model 7430, Ugo Basile, Comerio, Italy). The apparatus consisted of a transparent acrylic cage (35 cm × 23 cm × 20 cm) with a set of horizontal sensors to register locomotor activity and a set of vertical sensors to register rearing activity. Rats were placed into the center of cage and allowed to explore the apparatus for five minutes while the horizontal (locomotion) and vertical (rearing) movements were captured. Thereafter, rats were returned to their home cages. The open field was cleaned with 5% ethyl alcohol and allowed to dry between tests.

### Rotarod

Motor performance was tested using the ENV-577 M Rotarod system (Med Associates, Georgia, VT, USA). The time spent on the rotating rod was measured for each animal during 300-second trials. The speed of rod rotation was increased progressively from 3 to 30 rpm during the 300-second period. This test consisted of four consecutive days of testing sessions with two trials per day. The Rotarod system was cleaned with 5% ethyl alcohol between tests.

### Barnes maze

Spatial learning ability was examined in the Barnes maze using methods described previously [36]. The Barnes maze apparatus is an opaque disc 100 cm in diameter elevated 1 m above the floor by a tripod. Twenty holes, 8 cm in diameter, are located 9.5 cm from the perimeter, and a black escape box is placed under one of the holes. Distinct spatial cues are located all around the maze and are kept constant throughout the study. A fluorescent lamp (21 W) was placed above the center of the arena. A naive set of previously untested animals was positioned under an acrylic dome in the middle of the maze for one minute. Once the dome was lifted, the animal was able to orient to spatial cues and find the hole with the hidden burrow. The session ended when the rat entered the escape burrow or after five minutes had elapsed. If the rat did not enter the burrow by itself, it was gently guided and allowed to remain there for one minute before being returned to its home cage. This test consisted of one day of habituation session and five consecutive days of testing sessions with two trials per day. The location of the burrow was the same for each individual rat, but position was changed among rats in a group. The maze was cleaned with 5% ethyl alcohol to remove any odor trails. Rats are expected to reduce their latency to find the burrow, as a measure of spatial memory. Because the Barnes maze lacks strong reinforcement stimuli, rats may lack motivation and occasionally explore the maze after finding the target hole without entering. To rectify this confounding factor, we used the primary latency, which is the time elapsed to the first encounter of the escape burrow.

### Inhibitory avoidance

The inhibitory avoidance apparatus (Ugo Basile, Comerio, Italy) consisted of two acrylic compartments of the same size, one illuminated and the other dark, separated by an automatic door. The floor of both chambers was made of stainless steel rods and the floor of the dark chamber can be electrified. On the training session, animals were placed individually in the lighted compartment and the door was opened. Once the animal entered the dark compartment, the door was closed and an electric foot-shock (0.5 mA, 2 seconds in duration) was delivered through the grid floor. The animal was removed from the dark compartment and returned to its home cage immediately after receiving this shock. Twenty-four hours later the animals were subjected to the test session. Animals that failed to enter the dark compartment in 5 minutes were removed from the apparatus and assigned a ceiling score of 300 seconds. Long-term memory was assigned as the longer the animal stayed in the light chamber. The training session was performed on the day after the end of the Barnes maze test.

### Nuclear extracts

Nuclear extracts of each hippocampus were prepared as previously described [37]. Briefly, hippocampal structures were homogenized using a Dounce homogenizer in cold PBS supplemented with, 0.5 mM PMSF, 2.5 μg/ml leupeptin and 2.5 μg/ml antipain, and centrifuged at 4°C for 30 seconds at 12,000 × g. The supernatants (cytoplasmic extract) were reserved for immunoblot, and the pellets were resuspended in lysis buffer (10 mM HEPES pH 7.9, 1.5 mM MgCl_2_, 10 mM KCl, 0.1 mM EDTA, 0.5 mM PMSF, 2.5 μg/ml leupeptin, and 2.5 μg/ml antipain) and incubated on ice for 10 minutes. After addition of NP-40 (10%), samples were vigorously mixed and centrifuged for 30 seconds at 12,000 × g. Supernatant was discarded, and the pellet was resuspended in extraction buffer (20 mM HEPES pH 7.9, 25% glycerol, 1.5 mM MgCl_2_, 300 mM NaCl, 0.25 mM EDTA, 0.5 mM PMSF, 2.5 μg/ml leupeptin, 2.5 μg/ml antipain), incubated 20 minutes on ice, and centrifuged for 20 minutes at 12,000 × g at 4°C. The resulting supernatants containing nuclear proteins were stored at −80°C. Protein concentration was determined using the Bio-Rad (Richmond, CA, USA) colorimetric assay [38].

### Electrophoretic mobility shift assay

Electrophoretic mobility shift assay (EMSA) for NF-κB was performed by using the gel shift assay kit from Promega (Madison, WI, USA), as described previously [37]. ^32^P-NF-κB double-stranded consensus oligonucleotide probe (5’-AGTTGAGGGGACTTTCCCAGGC-3’; 25,000 cpm) and nuclear extracts (15 μg) were used. DNA-protein complexes were separated by electrophoresis through a 6% non-denaturing acrylamide:bis-acrylamide (37.5:1) gel in 0.53 Tris-borate/EDTA (TBE) for two hours at 150 V. Gels were vacuum dried and analyzed by autoradiography. For competition experiments, NF-κB and transcription initiation factor II (TFIID; 5’-GCAGAGCATATAAGGTGAGGTAGGA-3’) unlabeled double-stranded consensus oligonucleotide was included in 20-fold molar excess over the amount of ^32^P-NF-κB probe to detect specific and nonspecific DNA-protein interactions, respectively. Unlabeled oligonucleotides were added to the reaction mixture 20 minutes before the radioactive probe. Supershift assay incubations, using antibodies against different NF-κB subunits (p50, p52, cREL and RELA, 1:20 dilution), were also conducted according to the protocol of the manufacturer (Santa Cruz Biotechnology, Santa Cruz, CA, USA) before the incubation of nuclear extracts with the labeled oligonucleotide. Autoradiographs were visualized with a photodocumentation system DP-001-FDC (Vilber Lourmat, Marne la Vallée, France) and quantified in NIH ImageJ software (Bethesda, MD, USA). Several exposure times were analyzed to ensure the linearity of the band intensities.

### Immunoblotting

Electrophoresis was performed using a 10% polyacrylamide gel and the Bio-Rad mini-Protean III apparatus (Bio-Rad, Richmond, CA, USA). In brief, the proteins present in the hippocampus cytosolic (15 μg) and nuclear fractions (10 μg) were size separated in 10% SDS-PAGE (90 V). The proteins were blotted onto a nitrocellulose membrane (Bio-Rad, Richmond, CA, USA) and incubated with specific antibodies against RELA (sc-372; Santa Cruz Biotechnology, Santa Cruz, CA, USA). The Ponceau method of immunoblotting was used to ensure equal protein loading [39]. Proteins recognized by antibodies were revealed by the enhanced chemiluminescence (ECL) technique, following the instructions of the manufacturer (Amersham, Buckinghamshire, England). The membranes were exposed to x-ray film. To quantify the immunoblots, we used the NIH ImageJ software Bethesda, MA, USA). Several exposure times were analyzed to ensure the linearity of the band intensities. β-ACTIN antibody (sc-1616; Santa Cruz Biotechnology, Santa Cruz, CA, USA) was used as an internal control for the experiments. Results were expressed in relation to the intensity of β-ACTIN band.

### Multiplex analysis of cytokines and chemokines

Concentrations of IL-1α, IL-1β, TNF-α, IL-6, IFN-γ and RANTES were simultaneously measured in 25 μL of serum and extracts from homogenized hippocampus using a Milliplex MAP kit Rat Cytokine/Chemokine Magnetic Bead Panel (Millipore, Billerica, MA, USA) by following instructions of the manufacturer. Briefly, hippocampal tissue was homogenized in a buffer containing 150 mM NaCl, 0.05% Tween-20, 1 mM PMSF, 2.5 ug/ml antipain, 2.5 ug/ml leupeptin and 20 mM Tris-HCl (pH 7.5), and protein concentration was measured in each sample. Antibody immobilized beads were detected on a Luminex 100 xMAP technology machine (Austin, TX, USA). Standard curves were generated for each cytokine/chemokine using standards included in the kit diluted in kit matrix for serum samples and lysis buffer for tissue samples. The median fluorescence intensity for each analyte was calculated using a five-point logistic parameter curve, and normalized to the amount of protein in each sample. Concentrations of serum IL-1α and TNF-α, and tissue IL-1β and IL-10, were consistently below the level of detection of the assay. Therefore, we measured IL-10 and IL-1β levels in hippocampal extracts and TNF-α in serum by a conventional ELISA kit.

### ELISA

Hippocampal levels of IL-1β and IL-10, and serum levels of TNF-α were measured by ELISA using eBioscience (San Diego, CA, USA) kits, and hippocampal levels of brain-derived fa (BDNF) were measured using a Promega ELISA kit (Madison, WI, USA). To measure TNF-α, IL-1β and IL-10 levels, serum was isolated within 30 minutes after blood (5 ml) collection by centrifuging at 3,000 × g for 10 minutes, and cytosolic hippocampal extract was isolated by homogenizing hippocampi structures using a Dounce homogenizer in cold PBS supplemented with 0.5 mM PMSF, 2.5 μg/ml leupeptin and 2.5 μg/ml antipain, and centrifuging at 4°C for 30 seconds at 12,000 × g. To measure BDNF levels, hippocampus samples were prepared according to the instructions of the manufacturer. Briefly, the tissue was homogenized in lysis buffer (137 mM NaCl, 20 mM Tris HCl pH 8, 1% NP40, 10% glycerol, 0.5 mM PMSF, 2.5 μg/ml leupeptin, and 2.5 μg/ml antipain), then centrifuged for 2 minutes at 12,000 × g. The supernatants were stored at −80°C.

### RNA extraction and Real-Time RT-PCR

Total RNA was isolated using Trizol reagent (Invitrogen, Carlsbad, CA, USA) according to the instructions of the manufacturer. To avoid genomic DNA contamination, total RNA was treated with deoxyribonuclease I (DNase I) and RNase-free enzyme (Fermentas, Vilnius, Lithuania) before RT reactions. Quantification and purity of RNA samples were performed by measuring absorbance at 260 nm and 280 nm on a spectrophotometer. A 260/280 nm absorbance ratio ranging between 1.7 and 2.1 was considered an acceptable purity. cDNA was synthesized with reverse transcriptase from Promega (Madison, WI, USA) using random primers and following instructions of the manufacturer. Real-time RT-PCR analysis was performed with the 7500 Fast Real-Time PCR System (Applied Biosystems, Foster City, CA, USA). The PCR thermal cycling conditions were as follows: 95°C for 4 minutes, 40 cycles of 94°C for 45 seconds, 60°C for 45 seconds, 72°C for 45 seconds, and 80°C for 10 seconds (during which the fluorescence was measured), and final extension at 72°C for 7 minutes. Each reaction (performed in duplicate) included 3 μl of diluted (1:10) cDNA, 6 μl of 2x GoTaq qPCR Master Mix (Promega, Madison, WI, USA), primers (final concentration of 150 nM each), and sterile bi-distilled water up to a final volume of 12 μl. Amplicons that span over two or more exons were selected, whenever possible, to distinguish between amplification of mature mRNA from that of genomic DNA. Relative mRNA levels were calculated from cycle threshold values (Ct) using the REST method [40]. *Hprt* mRNA (hypoxanthine-guanine phosphoribosyltransferase) was used as an internal control for each individual sample. The mRNAs analyzed in this study were *iNos* and *Tlr4* (see Table 1 for primers).

**Table 1:** Primer sequences used in the real time RT-PCR

**Gene**	**Sequence**
*iNos* [GenBank: MN_0126113]	*Forward 5′* AAAATGGTTTCCCCCAGTTC *3′*
	*Reverse 5′* GTCGATGGAGTCACATGCAG *3′*
*Tlr-4* [GenBank: MN_019178.1]	*Forward 5′* GGATGATGCCTCTCTTGCAT *3′*
	*Reverse 5′* TGATCCATGCATTGGTAGGTAA *3′*
*Hprt* [GenBank: MN_012583.2]	*Forward 5′* CTCATGGACTGATTATGGACAG *3′*
	*Reverse 5′* GCAGGTCAGCAAAGAACTTATA *3′*

### Statistical analysis

Behavioral data of Barnes maze were represented as means ± SEM and analyzed using two-way ANOVA (treatment × trial time) with repeated measures (trial days) followed by Bonferroni *post hoc* test. In the inhibitory avoidance task, the heteroscedasticity of data and the use of the 300-second ceiling in test sessions required the use of nonparametric statistics. In this way, data were represented as medians and interquartile range and analyzed using Wilcoxon signed rank test (paired and non-parametric *t*-test). In order to verify the difference between groups, Kruskal-Wallis analysis of variance followed by Dunn’s multiple comparison test was performed. Data from mRNA analysis were analyzed by the pairwise fixed reallocation randomization test [40] using the relative expression software tool (REST) that incorporates the amplification efficiencies of the target and reference (normalization) genes to correct for differences between the two assays. All other data were analyzed using a one-way ANOVA followed by Newman-Keuls *post hoc* comparison of means (GraphPad Prism 5 software package). All results are expressed as mean ± SEM and *P* values < 0.05 were considered to reflect a statistically significant difference.

## Results

### Intermittent fasting ameliorates memory deficits caused by experimental systemic inflammation

The designs of experiments for tissue collection and behavioral testing are shown in Figure 1. Rats were maintained on either *ad libitum* (Control) or IF diets for 30 days and were then administered either vehicle (saline) or LPS intravenously. The animals of both groups were weighed before and after IF. The results showed that IF rats had increased body mass after 30 days of diet (Δ = 26.1 ± 3.0 g) but this increase was lower than that of the Control group (Δ = 57.1 ± 3.0 g) (*P < 0.05, Student’s *t*-test). Therefore, IF animals did gain body weight during the 30 days period, suggesting that IF did not interfere in the development of the animal. Such findings suggest that the animals did not consume twice their daily food intake on the *ad libitum* feeding day, which resulted in a reduced body weight gain.

**Figure 2:**
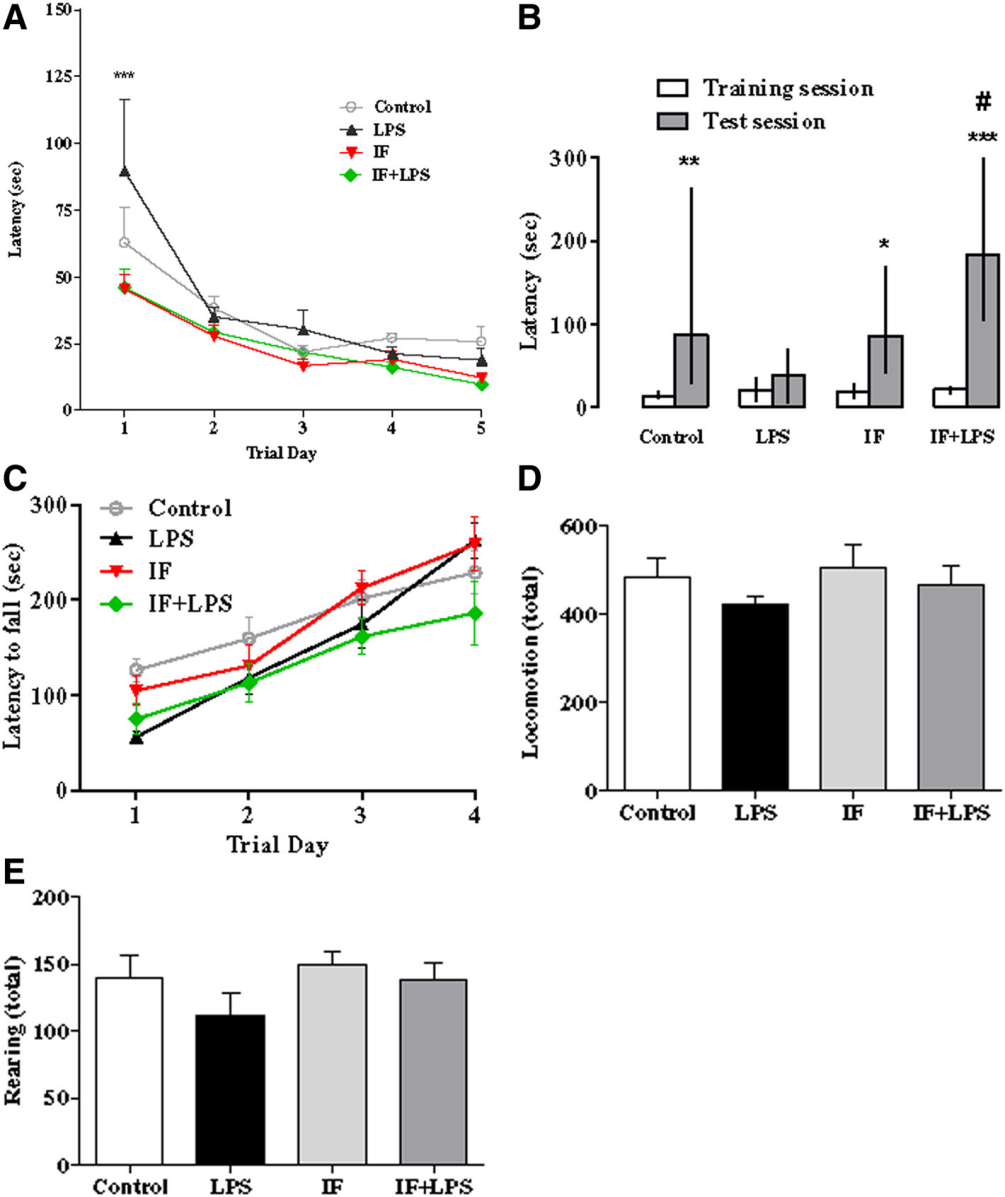
**Intermittent fasting (IF) prevents lipopolysaccharide (LPS)-induced memory impairment in the Barnes maze and inhibitory avoidance task. (A)** Barnes maze test results (***P < 0.001 versus IF and IF + LPS groups). Data are represented as the escape latency (time in seconds that the rat takes to find the correct hole containing the burrow). **(B)** Inhibitory avoidance test results (*P < 0.05 IF test session versus its training session; **P < 0.01 Control test session versus its training session; ***P < 0.001 IF + LPS test session versus its training session; #*P* < 0.05 IF + LPS versus LPS test sessions). Both tests showed that IF attenuated memory impairment elicited by LPS challenge. **(C)** Beginning 24 hours after LPS injection, mice were tested daily for motor performance on a rotarod. The latency to fall during the testing period (four days) are shown. **(D, E)** Results of the analysis of spontaneous locomotor activity in the electronic animal activity meter showing the total locomotion **(D)** and total rearing **(E)** 24 hours after LPS administration. N = 8 to 10 for each experimental group.

In the Barnes maze test, all rats were able to learn the location of the escape burrow, as latency to find the escape box decreased across the five blocks of daily observation in all groups (F_(4,194)_ = 20.31, *P* < 0.001 - Figure 2A). Moreover, treatment of animals with IF produced a significant difference in their performance in the maze (F_(3,194)_ = 4.43, *P* < 0.01). Bonferroni post-test analysis indicated significantly shorter latencies for IF and IF + LPS groups when compared to animals injected with LPS (*P* < 0.001) at trial one. As all animals experienced a previous day of habituation, these data suggest that IF increased their capacity to perform the learning and memory task in the presence of the inflammatory insult. In addition, LPS treated animals did not acquire the information (impaired learning) and, because of this, in the first evaluation day, they had a worse performance. However, IF + LPS animals, submitted to the same protocol, showed better performance in the first evaluation day, suggesting that IF was effective in protecting against LPS-induced learning impairment.

In the inhibitory avoidance test, no significant differences were detected between groups during the training session (Figure 2B). Results of long-term memory in the test session 24 hours after the training session showed a statistically significant difference in performance between groups. Significantly greater latencies in test sessions were observed in Control group (87.4 sec (11.7/300.0 seconds), n = 8, *P* < 0.05), IF group (85.6 sec (15.3/298.0 seconds), n = 10, *P* < 0.01), and IF + LPS group (183.1 sec (82.1/300.0 seconds), n = 9, *P* < 0.001) when compared to the respective latencies in training session, indicating that these animals learned and memorized the task. Concerning the LPS group, there was no significant difference between the test session (38.6 sec (4.7/75.0 seconds), n = 9) and training session latency (LPS Control group = 20.7 (5.6/36.9 seconds), n = 9), indicating that this group did not learn the task. These findings confirmed previous data reporting the long-lasting effects induced by LPS in animal brains [25,41]. In addition, they showed that IF ameliorates the long-term memory consolidation deficit induced by LPS.

Although the comparable performances during the learning phase argued against any interference due to LPS-induced motor function impairment, change in motor skills was evaluated by using electronic animal activity meter and rotarod apparatus. Results showed no significant difference in the locomotor performance (locomotion and rearing) at 24 hours of LPS treatment (LPS and IF + LPS groups) when compared to Control and IF groups (Figure 2D and E). Similarly, performances of LPS-treated groups were indistinguishable from Control and IF groups regarding the latency to fall from a rotating rod (Figure 2C). Collectively, our results suggest that LPS causes cognitive impairment, specifically a deficit in long-term memory retention, which can be ameliorated by IF pretreatment.

### Intermittent fasting modulates NF-κB transcription factor

**Figure 3:**
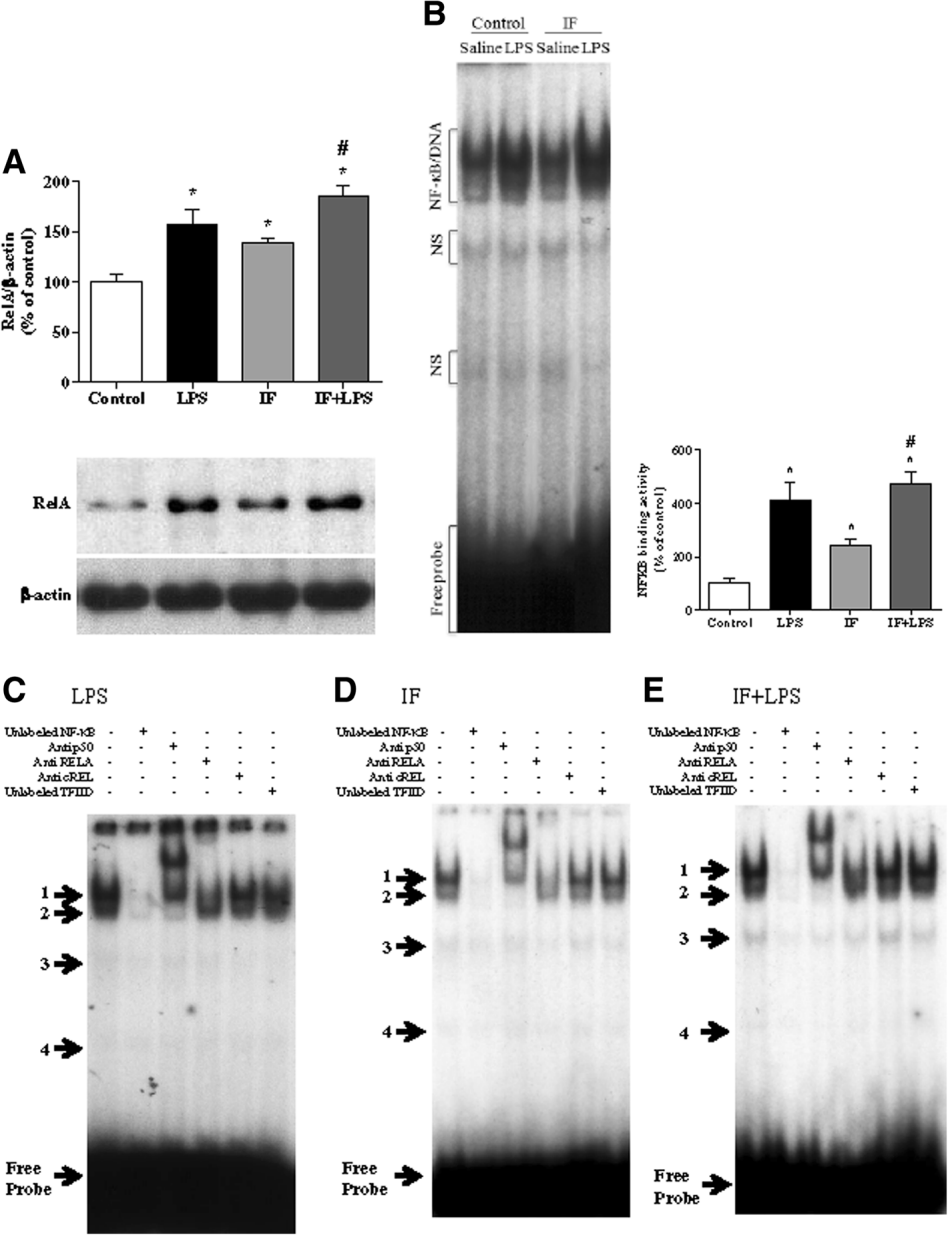
**Lipopolysaccharide LPS) treatment and intermittent fasting (IF) increase the NF-κB nuclear translocation and binding activity.** Electrophoretic mobility shift assay (EMSA) and Western blot were performed using nuclear extract (10 μg) from rat hippocampus. **(A)** Western blot results using antibodies to RELA and β-ACTIN showed that IF and LPS treatment with or without IF increased the nuclear translocation of RELA. **(B)** EMSA showed that LPS treatment increased the NF-κB binding activity in rats submitted or not to IF protocol. IF by itself also increased NF-κB binging activity. Competition and supershift assays of NF-κB activation by LPS treatment **(C)**, IF **(D)** and IF with LPS treatment **(E)** were carried out. Competition studies were performed using the nuclear extract in the absence or presence of unlabeled specific (NF-κB consensus sequence, 20-fold molar excess) or nonspecific (TFIID consensus sequence, 20-fold molar excess) oligonucleotide, as indicated. Supershift assays were performed with the same nuclear extract incubated in the absence or presence of antibodies (1:20 dilution) against subunits p50, RELA, and cREL as indicated. Antibodies were added 20 minutes prior to addition of the radiolabeled NF-κB consensus oligonucleotide. The positions of specific NF-κB/DNA and non-specific (NS) binding complexes are indicated. *P < 0.05 versus Control; #*P* < 0.05 versus IF. N = 5 for each experimental group.

Next, we investigated whether the IF could modulate the activities of NF-κB with or without exposure to LPS. To evaluate the nuclear translocation of NF-κB, we examined nuclear protein levels by Western blot using RELA specific polyclonal antibody. Figure 3A shows that nuclear translocation of NF-κB was significantly increased by IF itself and after LPS treatment in the presence or absence of IF. To verify the DNA-binding activity of NF-κB, EMSA was performed with nuclear proteins isolated from rat hippocampus. Results showed that the binding activity of NF-κB was increased by LPS treatment and also by IF and IF + LPS treatment (Figure 3B). However, NF-κB activation and RELA translocation induced by LPS in both groups (LPS and IF + LPS) were similar and they were also higher than the IF group, suggesting a different pattern of response. Interestingly, hippocampal nuclear extracts of all groups analyzed exhibited a similar pattern of four DNA/protein complexes (Figure 3C, D and E). The upper complexes 1 and 2 were displaced by an excess of unlabeled NF-κB, but not TFIID double-stranded oligonucleotide consensus sequence, demonstrating the specificity of NF-κB/DNA binding interaction (Figure 3C, D and E). The lower complexes were less efficiently displaced by unlabeled NF-κB probe. Supershift analysis indicated that the antibody against the subunit RELA was able to shift DNA/protein interaction present in complex 1. The antibody against the subunit p50 shifted complex 2 and induced a partial decrease in complex 1 (Figure 3C, D and E). The presence of antibodies against the subunits cREL (Figure 3C, D and E) and p52 (data not shown) did not affect DNA-protein complexes. Taken together, these results indicated that p50/RELA heterodimers and p50/p50 homodimers were included in ^32^P-NF-κB/protein complexes 1 and 2, respectively. Complexes 3 and 4 were not displaced by the antibodies and were not considered related to NF-κB family.

### Intermittent fasting reduces levels of hippocampal Tlr-4 and iNos mRNAs

**Figure 4:**
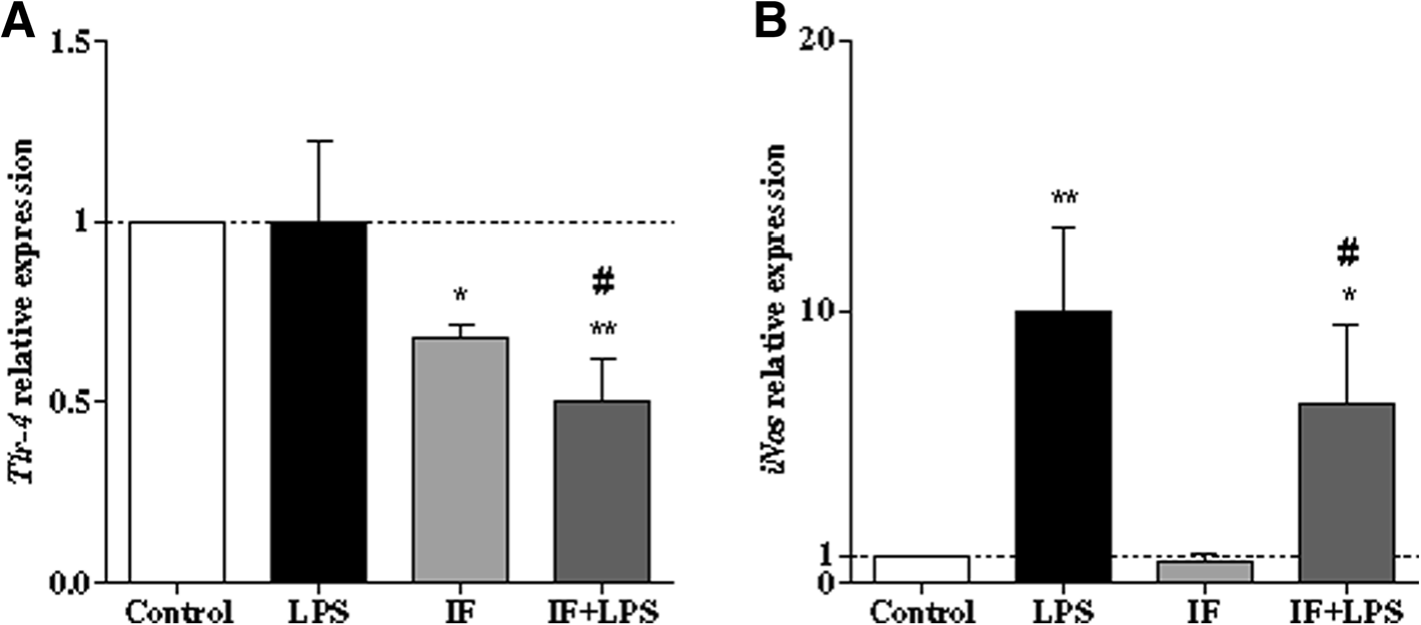
**Intermittent fasting reduces toll-like receptor 4 (TLR4) and attenuates lipopolysaccharide (LPS)-induced synthase (iNOS) up-regulation in the hippocampus. (A)** Levels of *Tlr-4* mRNA (*P = 0.008 versus Control; **P = 0.004 versus Control; #*P* = 0.016 versus LPS). **(B)** Levels of *iNos* mRNA (**P = 0.015 versus Control; *P = 0.013 versus Control; #*P* = 0.045 versus LPS). The mRNA levels were measured by quantitative RT-PCR in samples from hippocampus. N = 5 for each experimental group.

Because TLR4 and iNOS mediate adverse effects of systemic inflammation/LPS on hippocampal plasticity [18,42,43], we measured levels of mRNAs encoding TLR4 and iNOS in hippocampal samples from rats in the four groups. After normalization with the housekeeping *Hprt* mRNA levels, the expression levels were presented as a relative fold change compared with the Control group. The *Tlr-4* mRNA levels were significantly lower in hippocampi of rats in the IF and IF + LPS groups compared to the Control group (Figure 4A). *Tlr-4* mRNA levels were also significantly lower in the IF + LPS group compared to the LPS group (Figure 4A). Levels of *iNos* mRNA increased sharply after LPS injection with or without IF, by 9.95-fold and 6.60-fold respectively. The *iNos* mRNA levels were significantly lower in the IF + LPS group compared to the LPS group (Figure 4B).

### IF suppresses LPS-induced accumulation of inflammatory proteins in the hippocampus

**Figure 5:**
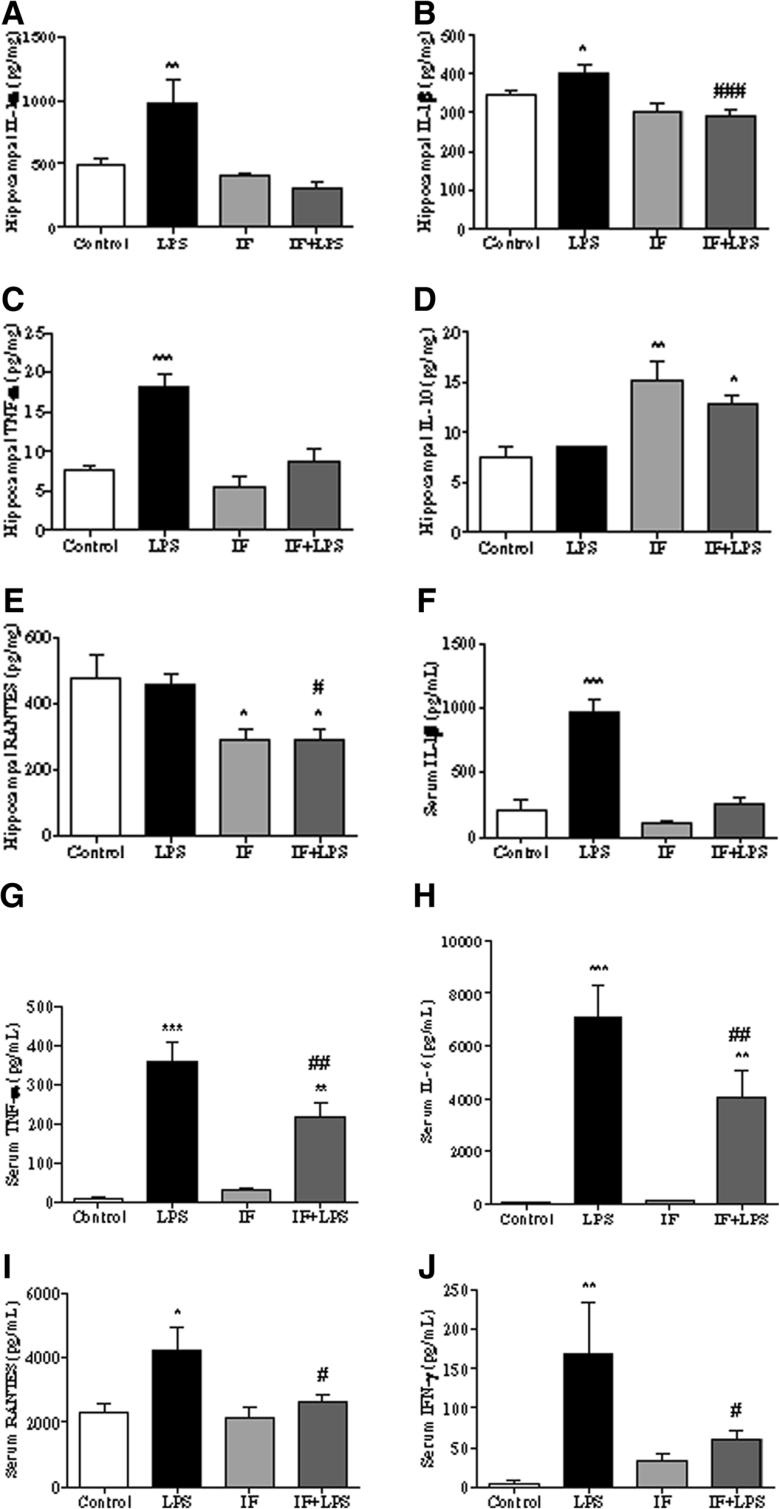
**Intermittent fasting (IF) inhibits lipopolysaccharide LPS)-induced increases in inflammatory mediators in the hippocampus and serum.** Levels of hippocampal IL-1α **(A)**, IL-1β **(B)**, TNF-α **(C)**, IL-10 **(D)** and RANTES **(E)**, and levels of serum IL-1β **(F)**, TNF-α **(G)**, IL-6 **(H)**, RANTES **(I)** and IFN-γ **(J)** were measured by multiplex or ELISA (*P < 0.05 versus Control; **P < 0.01 versus Control; ***P < 0.001 versus Control; #*P* < 0.05 versus LPS; ##*P* < 0.01 versus LPS and ###*P* < 0.001 versus LPS. N = 5 to 10 for each experimental group.

In view of the important role played by cytokines and chemokines in LPS-induced neuroinflammation [23,24,26,44], we investigated the levels of several pro-inflammatory cytokines and chemokines (IL-1α, IL-1β, TNF-α, IL-6, IFN-γ, and RANTES) and the anti-inflammatory cytokine IL-10 in the periphery (serum) and in the hippocampus, a brain region in which neuroinflammation occurs in response to brain injury and systemic inflammation [45,46]. Multiplex and ELISA immunoassay showed that LPS caused a significant increase of IL-1α (Figure 5A), IL-1β (Figure 5B), and TNF-α (Figure 5C) in the hippocampus, and IL-1β (Figure 5 F), TNF-α (Figure 5G), IL-6 (Figure 5H), RANTES (Figure 5I), and IFN-γ (Figure 5 J) in the serum. Remarkably, IF significantly attenuated or completely prevented the LPS-induced increases in all of the pro-inflammatory cytokines in hippocampus and serum (Figure 5). In addition, levels of RANTES were significantly lower (Figure 5E) and levels of the anti-inflammatory cytokine IL-10 were significantly elevated (Figure 5D) in the hippocampi of rats in the IF and IF + LPS groups compared to the Control and LPS groups.

### IF prevents LPS-induced reduction of hippocampal BDNF levels

**Figure 6:**
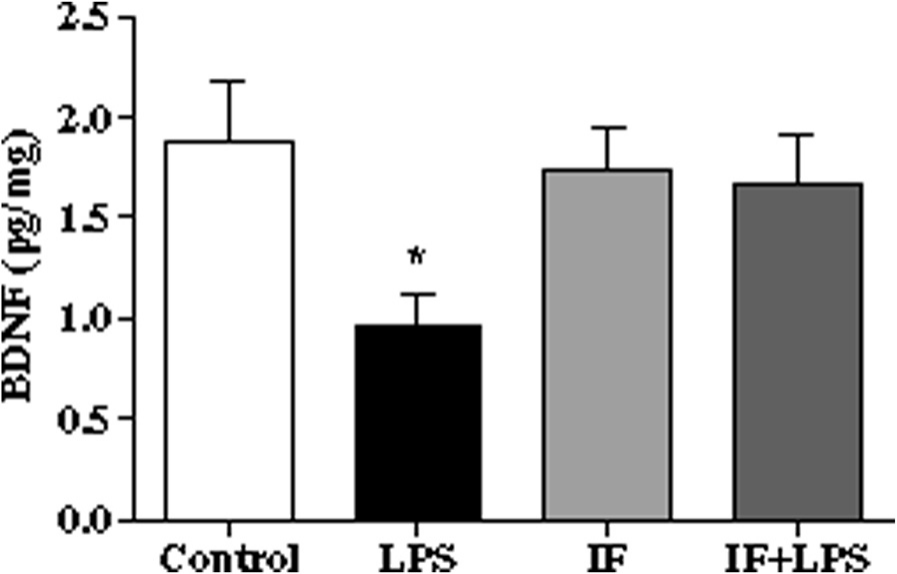
**Intermittent fasting (IF) prevents lipopolysaccharide (LPS)-induced depletion of hippocampal brain-derived neurotrophic factor (BDNF).** Levels of BDNF were measured in hippocampal extracts by ELISA (*P < 0.05 versus Control, IF, and IF + LPS groups. N = 10 for each experimental group.

BDNF plays an important role in memory and neuroprotection [47]. In our model, we found by ELISA immunoassay that LPS treatment significantly reduced hippocampal BDNF levels when compared to all other groups (Figure 6). This was not observed in the IF + LPS group (Figure 6), suggesting that IF can prevent the reduction of hippocampal BDNF levels caused by systemic inflammation.

## Discussion

A wide array of health benefits of IF have been identified in animal models and human studies [48]. In contrast, no studies evaluating the effect of IF in neuroinflammatory processes induced by LPS administration have been conducted to date. Based on studies showing that IF can protect neurons against degeneration in animal models of injury that involve local inflammatory processes (stroke and seizures) and an AD model [31,32,34], and evidence that sepsis can cause cognitive impairment in human subjects [49], we asked whether IF might modify the adverse effects of systemic inflammation on cognitive function. Using LPS to elicit an immune response, we observed significant impairment of memory performance in Barnes maze and inhibitory avoidance tests that was counteracted by IF.

The Barnes circular platform was chosen as a spatial memory paradigm [50]. This task is less stressful than the water maze [51] and is based on the natural tendency of mice to explore and escape through holes [52,53]. We observed that, despite all groups having learned the burrow location across successive days, there was a significant effect of IF in improving the performance of LPS-treated rats compared to LPS-treated rats on the Control *ad libitum* diet. The step-through inhibitory avoidance test is based on the natural dark-seeking behavior of rats and evaluates long-term, declarative memory. It is a fear-motivated task in which the rat refrains from stepping through a door to an apparently safer dark compartment previously linked to a punishment [54,55]. Our results showed that animals treated with LPS without IF did not remember the aversive stimulus (foot-shock), unlike the animals previously subjected to IF. The similar motor performance exhibited by all groups (Control, IF, LPS and IF + LPS) during the learning phase in Barnes and inhibitory avoidance behavioral assays as well as results from electronic motor activity meter and rotarod apparatus confirmed that the IF effect against LPS-induced memory impairment is not linked to changes in general activity or motor performance differences. Therefore, data here showed that IF effectively prevented the memory deficit induced by systemic inflammatory bacterial LPS administration, suggesting a potential application of such diets to those with or at risk for cognitive impairment.

Cognitive decline is prominent in AD, and also occurs in other disorders in which neuroinflammation is believed to play a prominent role [44]. Thus, the next step was to evaluate the effects of IF on neuroinflammatory processes induced by LPS administration. Confirming our previous results [35,56], LPS activates nuclear RELA translocation and NF-κB binding activity increasing pro-inflammatory genes within two hours in rat hippocampus. Although IF also induced an increase in NF-κB activation and nuclear RELA translocation, the LPS response in the presence or absence of IF was higher when compared to the IF group. Based on the evidence that NF-κB nuclear extracts of all groups analyzed presented a similar pattern of four DNA/protein complexes, we consider that IF induced different changes in the expression of NF-κB target genes involved in synaptic plasticity and cell survival linked to a cell specific NF-κB activation when compared to LPS, since IF is mediating a shift in LPS-induced pro-inflammatory gene expression towards an anti-inflammatory signaling cascade. In fact, there is evidence supporting a dual role of NF-κB in neurodegenerative diseases in the central nervous system (CNS); activation of NF-κB in neurons promotes their survival, whereas activation in glial and immune cells mediates pathological inflammatory processes [16]. Confirming this hypothesis, we found that levels of multiple markers of neuroinflammation induced by LPS via NF-κB activation were reduced in rats on the IF diet compared to those on the Control diet, including iNOS, pro-inflammatory cytokines, and the LPS receptor TLR4. In addition, our previous results showed that the chronic unpredictable stress (CUS) protocol also induced an increase in NF-κB activation and nuclear RELA translocation, but different from IF, CUS augmented the effects of LPS on NF-κB activation and nuclear RELA translocation in hippocampus, as well as the effects of LPS on expression of the pro-inflammatory genes [56]. Therefore, while CUS-induced sensitization of microglia reactivity (‘priming’ stress effects) enhances inflammation [57], IF is probably acting by engaging an adaptive cellular stress-response signaling pathways in neurons, thus decreasing LPS response [58].

TLR4 is expressed in astrocytes, microglia and neurons wherein its activation can adversely affect neuronal plasticity and survival in brain injury settings [21,59]. In addition, TLR4 signaling inhibits neurogenesis [60], which may contribute to the impairment of cognitive function by neuroinflammation. Here, we showed that IF inhibited LPS-induced production of pro-inflammatory mediators including TNF-α, IL-1β, IL-6, IL-1β, RANTES, and *iNos*. These effects of IF are indicative of decreased activation of inflammatory cascades linked to NF-κB mediating pro-inflammatory response [14,61]. In fact, it is known that high concentrations of NO, unlike low concentrations [42,43], may promote excitotoxicity, which may contribute to cognitive impairment [43,62-64]. Our results showed that IF was able to mitigate the induction of *iNos* expression by LPS. This result may indicate that the improved memory performance observed in the IF + LPS group may be related to a reduction of hippocampal *iNos* and consequently the pathological levels of NO, which could generate a cognitive deficit.

We also found that IF increases levels of IL-10, a cytokine that is known to counteract NF-κB pro-inflammatory signaling. The pro-inflammatory cytokine TNF-α plays a central role in the onset and maintenance of inflammation [65], while IL-10 is a prototypical anti-inflammatory cytokine in that it inhibits production of inflammatory cytokines and chemokines such as IL-1β, IL-6, TNF-α, IFN-γ and RANTES [66,67]. IL-10 also acts as a natural antagonist of TNF-α by inhibiting the NF-κB inflammatory signaling through the preservation of the inhibitory protein IκB [68]. Therefore, IF simultaneously suppresses pro-inflammatory signaling pathways and activates at least one anti-inflammatory pathway.

Neuroinflammatory conditions such as traumatic brain injury, AD, Down syndrome and aging are frequently associated with cognitive impairment [69-71], and there is considerable evidence for an association between cytokine expression in the brain and cognitive deficits, including memory deficits [72-74]. For instance, it has been shown that TNF-α and IL-6 are critical for neuroinflammation-induced memory impairment [75-77]. Also, it is well defined that acute inflammation induced by LPS or IL-1β injection causes memory deficits [28,78-82]. Accordingly, it was previously shown that hippocampal IL-1β overexpression impairs contextual and spatial long-term memory [74], while IL-10 inhibits LPS-induced sickness behavior and tribulations in hippocampal-dependent memory [73].

Previous studies have shown that caloric restriction and IF reduce levels of circulating pro-inflammatory IL-1β and TNF-α, and increase anti-inflammatory IL-10 levels in animal models and human subjects [83-85]. In the brain, IL-10 may inhibit the development and progression of chronic neurodegenerative diseases. For example, the onset of neuroinflammation and progression of prion disease was accelerated in mice deficient in IL-10 [86]. In the present study, IF exerted an anti-inflammatory effect by increasing hippocampal IL-10 levels, and by preventing LPS-induced increases in IL-1α, IL-1β, TNF-α, and RANTES levels. IF also inhibited LPS-induced increases in serum TNF-α, IL-6, RANTES and IFN-γ, suggesting that IF anti-inflammatory effect is not restricted to the CNS.

Finally, our findings suggest that IF can sustain neurotrophic support for hippocampal neurons in the setting of systemic inflammation. The hippocampus has been demonstrated to be a major brain structure involved in spatial learning [87]. It was previously demonstrated that LPS reduces hippocampal mRNA levels of BDNF, a neurotrophin that plays an important role in learning and memory processes [88]. Accordingly, we found that LPS administration caused a reduction in BDNF levels in the hippocampus of rats on the Control diet, but not in rats on the IF diet. Thus, in addition to suppressing the local (hippocampal) and systemic inflammatory responses to LPS, IF prevented the reduction of BDNF levels in this animal model of systemic inflammation. Because BDNF plays critical roles in hippocampal synaptic plasticity and cognitive function, it is likely that maintenance of BDNF signaling contributes to the beneficial effects of IF on cognitive function in LPS-treated rats. Previous studies have provided evidence that BDNF is a key target of NF-κB involved in NMDA receptor-mediated cell survival signaling [89-92] and IF has been shown to activate glutamate signaling cascade in the CNS [58]. The ability of IF to suppress inflammatory processes and sustain hippocampal BDNF levels in the face of systemic inflammation, suggests that IF interventions should be evaluated in studies of human subjects at risk for or suffering from neurological conditions involving neuroinflammation.

Interleukin - (IL)-1α, Interferon - (IFN)-γ, Non-Steroidal Anti-Inflammatory Drugs –NSAIDs, Tumor Necrosis Factor -(TNF), Nuclear factor-κB - (NF-κB), Interleukin (IL)-1β, Brain-Derived Neurotrophic Factor – (BDNF) inducicble nitric oxide synthase (iNOS).

## Competing interests

The authors declare that they have no competing interests.

## Authors’ contributions

Conceived and designed the experiments: ARV, CS, TV and EMK. Performed the experiments: ARV and LMY. Analyzed the data: ARV, CS, TV, HSB, MPM and EMK. Contributed reagents/materials/analysis tools: ARV, CS, TV, HSB, EMK and SKPC. Wrote the paper: ARV, CS, MPM and EMK. All authors read and approved the final manuscript.
